# Synthesis, Preclinical Evaluation, and a Pilot Clinical PET Imaging Study of ^68^Ga-Labeled FAPI Dimer

**DOI:** 10.2967/jnumed.121.263016

**Published:** 2022-06

**Authors:** Liang Zhao, Bo Niu, Jianyang Fang, Yizhen Pang, Siyang Li, Chengrong Xie, Long Sun, Xianzhong Zhang, Zhide Guo, Qin Lin, Haojun Chen

**Affiliations:** 1 Department of Nuclear Medicine and Minnan PET Center, The First Affiliated Hospital of Xiamen University, Xiamen, China;; 2 Department of Radiation Oncology, The First Affiliated Hospital of Xiamen University, Xiamen, China;; 3 School of Medicine, Xiamen University, Xiamen, China;; 4 State Key Laboratory of Molecular Vaccinology and Molecular Diagnostics and Center for Molecular Imaging and Translational Medicine, School of Public Health, Xiamen University, Xiamen, China; and; 5 Fujian Provincial Key Laboratory of Chronic Liver Disease and Hepatocellular Carcinoma, Xiamen, China

**Keywords:** fibroblast activation protein, cancer-associated fibroblasts, FAPI dimer, patient-derived xenografts, PET imaging

## Abstract

Cancer-associated fibroblasts (CAFs) are crucial components of the tumor microenvironment. Fibroblast activation protein (FAP) is overexpressed in CAFs. FAP-targeted molecular imaging agents, including the FAP inhibitors (FAPIs) 04 and 46, have shown promising results in tumor diagnosis. However, these molecules have a relatively short tumor-retention time for peptide-targeted radionuclide therapy applications. We aimed to design a ^68^Ga-labeled FAPI dimer, ^68^Ga-DOTA-2P(FAPI)_2_, to optimize the pharmacokinetics and evaluate whether this form is more effective than its monomeric analogs. **Methods:** ^68^Ga-DOTA-2P(FAPI)_2_ was synthesized on the basis of the quinoline-based FAPI variant (FAPI-46), and its binding properties were assayed in CAFs. Preclinical pharmacokinetics were determined in FAP-positive patient-derived xenografts using small-animal PET and biodistribution experiments. The effective dosimetry of ^68^Ga-DOTA-2P(FAPI)_2_ was evaluated in 3 healthy volunteers, and PET/CT imaging of ^68^Ga-FAPI-46 and ^68^Ga-DOTA-2P(FAPI)_2_ was performed on 3 cancer patients. **Results:** ^68^Ga-DOTA-2P(FAPI)_2_ was stable in phosphate-buffered saline and fetal bovine serum for 4 h. The FAPI dimer showed high affinity and specificity for FAP in vitro and in vivo. The tumor uptake of ^68^Ga-DOTA-2P(FAPI)_2_ was approximately 2-fold stronger than that of ^68^Ga-FAPI-46 in patient-derived xenografts, whereas healthy organs showed low tracer uptake and fast body clearance. The effective dose of ^68^Ga-DOTA-2P(FAPI)_2_ was 1.19E−02 mSv/MBq, calculated using OLINDA. Finally, the PET/CT scans of the 3 cancer patients revealed higher intratumoral uptake of ^68^Ga-DOTA-2P(FAPI)_2_ than of ^68^Ga-FAPI-46 in all tumor lesions (SUV_max_, 8.1–39.0 vs. 1.7–24.0, respectively; *P* < 0.001). **Conclusion:** ^68^Ga-DOTA-2P(FAPI)_2_ has increased tumor uptake and retention properties compared with ^68^Ga-FAPI-46, and it could be a promising tracer for both diagnostic imaging and targeted therapy of malignant tumors with positive expression of FAP.

## Visual Abstract

**Figure:**
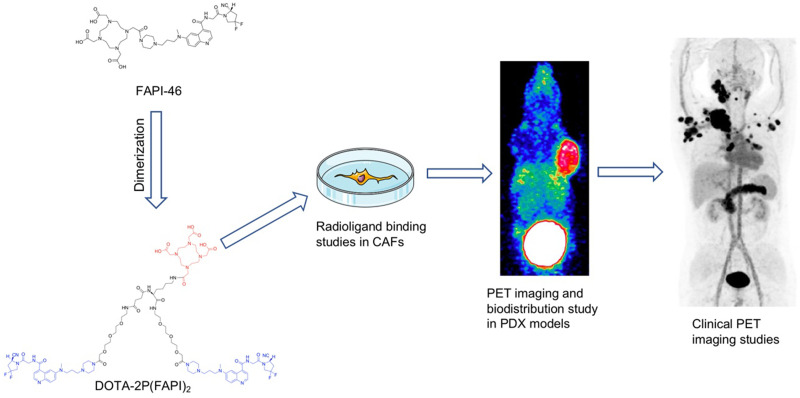

Cancer-associated fibroblasts (CAFs) are crucial components of the tumor microenvironment and can constitute over half the mass in various types of tumor. According to previous reports, CAFs play important roles in tumor growth, immune suppression, and cancer invasion (1*,*^2^). Thus, CAFs may be promising targets for tumor diagnosis and therapy. Fibroblast activation protein (FAP) is overexpressed in the CAFs of numerous epithelial carcinomas and weakly expressed in healthy tissues, therefore representing an attractive target for cancer research. The past few years have witnessed an expansion of research on FAP-targeted molecular imaging in tumor diagnosis (3–5).

Recently, there has been growing application of FAP targeting, from diagnostic imaging to peptide-targeted radionuclide therapy (PTRT) (6–10). However, the reports on PTRT are based mainly on peptides of the FAP inhibitors (FAPIs) 04 and 46, which showed a relatively short tumor-retention time in preclinical models and human subjects (10–12). Another FAPI variant, FAP-2286, has been studied in PTRT to improve tumor-retention time (13). Moreover, mouse models used to evaluate the pharmacokinetics of FAPI variants were cancer cell–derived xenografts in previous research (7*,*^10^*,*^11^). When xenotransplanted, cancer cell-derived xenografts adequately recruit mouse fibroblasts during tumor growth; thus, they are highly suitable for direct tracer comparisons (14). However, patient-derived xenografts (PDXs), established by direct implantation of fresh surgical tissue fragments into immunodeficient mice, could be more attractive because they retain the tumor environment and molecular signature of the corresponding parental tumor compared with that of cancer cell-derived xenografts (15). However, the potential of PDXs for PET imaging of CAFs has rarely been investigated.

The polyvalency effect has been applied to develop multimeric peptides to enhance the tumor-targeting efficacy of the tracers and improve the quality of in vivo imaging (16*,*^17^). Moreover, adding an amphiphilic polyethylene glycol linker (PEGylation) has been widely used to improve the in vivo kinetics of various pharmaceuticals (16*,*^18^). In the present study, we designed and synthesized a novel FAPI dimer with 2 mini-PEG spacers (11-amino-3,6,9-trioxaundecanoic acid, with 3 ethylene oxide units) between the 2 FAPI motifs in the homodimeric peptides, denoted as DOTA-2P(FAPI)_2_. The novel dimeric FAP-targeted molecule was labeled with the positron-emitting radionuclide ^68^Ga (^68^Ga-DOTA-2P(FAPI)_2_) for PET imaging. We present the results of ^68^Ga-DOTA-2P(FAPI)_2_ testing in PDX models, healthy volunteers, and cancer patients. We hypothesized that the dimeric FAPI is more effective than monomeric analogs in terms of tumor uptake and tumor-retention time.

## MATERIALS AND METHODS

### Chemistry and Radiochemistry

The vender information concerning chemicals, cells, reagents, synthesis procedure, high-performance liquid chromatography, liquid chromatography-mass spectrometry, and the flow diagram of DOTA-2P(FAPI)_2_ is provided in the supplemental materials and Supplemental Figure 1 (supplemental materials are available at http://jnm.snmjournals.org).

FAPI variants were radiolabeled by adjusting a mixture of 50 μg (56.4 nmol) of FAPI-46 or 50 μg (25.3 nmol) of DOTA-2P(FAPI)_2_, and 4 mL of ^68^Ga solution (1.3 GBq in 0.6 M HCl) to pH 3.3–3.6 with 1 mL of sodium acetate (0.25M in water; total volume of reaction, 5 mL). After being heated to 100°C for 15 min, the product was isolated by a C18 Sep-Pak cartridge (WAT020515; Waters) using ethanol (0.5 mL) as the eluent. Quality control of the radiosynthesis was performed using ultraviolet and radio–high-performance liquid chromatography (details presented in the supplemental materials).

The radiolabeled compound was incubated in phosphate-buffered saline and fetal bovine serum (FBS) at 37°C for 1, 2, and 4 h to measure the in vitro stability. Then, 0.5 mL of acetonitrile was added to remove plasma proteins from the serum after the sample was centrifuged at 1, 2, and 4 h. Finally, the radiochemical purities were analyzed using radio–high-performance liquid chromatography.

### PDX Model Establishment

Written informed consent was obtained from all patients, and the research protocol was approved by the Clinical Research Ethics Committee of the First Affiliated Hospital of Xiamen University (ID KYZ-2017-001). All animal care and experimental procedure were reviewed and approved by the Animal Care and Use Committee of the Xiamen University Laboratory Animal Center (ID XMULAC20170063). The establishment of PDX models was based on our previous protocols, detailed in the supplemental materials (19).

### Western Blot and Histopathologic Staining

Western blot analysis was performed in CAFs and the Huh7 cell line to select cells expressing FAP. CAFs or Huh7 cells were cultured in RPMI 1640 or Dulbecco modified Eagle medium containing 10% FBS at 37°C in 5% CO_2_. Western blotting and histopathologic staining were performed as described in the supplemental materials according to our previous protocol (20).

### Radioligand Binding Studies

Radioligand binding studies included cell uptake, cell uptake blocking, and FAP binding assay. CAFs expressing FAP were seeded in 24-well plates with 1640 medium containing 10% FBS and cultivated for 48 h to a density of approximately 80% before the experiments. The medium was replaced with 1640 medium without FBS. ^68^Ga-DOTA-2P(FAPI)_2_ or ^68^Ga-FAPI-46 or ^68^Ga-DOTA-2P(FAPI)_2_ with 11.3 nmol of unlabeled FAPI-46 (for the blocking experiment) was added to the 24-well plates and incubated for the scheduled times (10, 30, 60, 90, and 120 min). The FAP-binding assays were performed by simultaneous exposure to unlabeled FAPI variants (1.27 × 10^−4^ to 10^−13^ M for ^68^Ga-DOTA-2P(FAPI)_2_; 2.83 × 10^−4^ to 10^−13^ M for ^68^Ga-FAPI-46) and radiolabeled compounds for 60 min. The inhibitory concentration of 50% was calculated by fitting the data by nonlinear regression using GraphPad Prism. In each step of the experiments, the cells were washed twice with 1 mL of phosphate-buffered saline. Finally, CAFs were lysed with 0.5 mL of 1 M NaOH for radioactivity counting (counts per minute), examined in a γ-counter. Each independent experiment was repeated 3 times.

### PET Imaging and Biodistribution Study in Hepatocellular Carcinoma (HCC)-PDX Models

The products of ^68^Ga-DOTA-2P(FAPI)_2_ and ^68^Ga-FAPI-46 were diluted to a concentration of 74 MBq/mL, and 7.4 MBq (bout 0.1 mL) of ^68^Ga-DOTA-2P(FAPI)_2_ or ^68^Ga-FAPI-46 were intravenously injected into HCC-PDXs (*n* = 3 for each group). All PET scans were conducted using an Inveon small-animal PET scanner (Siemens Preclinical Solution). Dynamic and static PET imaging procedures are provided in the supplemental materials.

In the biodistribution study, the products of ^68^Ga-DOTA-2P(FAPI)_2_ and ^68^Ga-FAPI-46 were diluted to a concentration of 14.8 MBq/mL. HCC-PDX mice were injected with the same batch of 1.48-MBq ^68^Ga-DOTA-2P(FAPI)_2_ and killed at different times (1 and 4 h after injection; *n* = 3 for each time point). The main organs and tumors were isolated, weighed, and analyzed. The biodistribution in the ^68^Ga-FAPI-46 group (1.48 MBq) and the blocking group (^68^Ga-DOTA-2P(FAPI)_2_ [1.48 MBq] with 30 nmol of unlabeled FAPI-46) was also evaluated for comparison. The radioactivity (counts per minute) was measured with a γ-counter.

### PET Imaging in Healthy Volunteers and Cancer Patients

The clinical study was registered at ClinicalTrials.gov (NCT04941872). The Clinical Research Ethics Committee of the First Affiliated Hospital of Xiamen University approved the study, and all subjects gave written informed consent. Safety data were collected before and 4 h after injection of ^68^Ga-DOTA-2P(FAPI)_2_, including vital signs (blood pressure, heart rate, respiratory frequency, and temperature) and adverse events. The scan and reconstruction procedures are presented in the supplemental materials according to a previously described protocol (21). Time–activity curve fitting and subsequent dose calculations were performed using OLINDA/EXM, version 1.1 (22).

### Statistical Analysis

All quantitative data are expressed as mean ± SD. All statistical analyses were conducted using SPSS, a statistical analysis software program (version 22.0; IBM). One-way ANOVA, Student *t-*testing, and Wilcoxon matched-pairs signed-rank testing were used to compare means. Statistical significance was set at a *P* value of less than 0.05.

## RESULTS

### Synthesis and Radiolabeling

**FIGURE 1.**
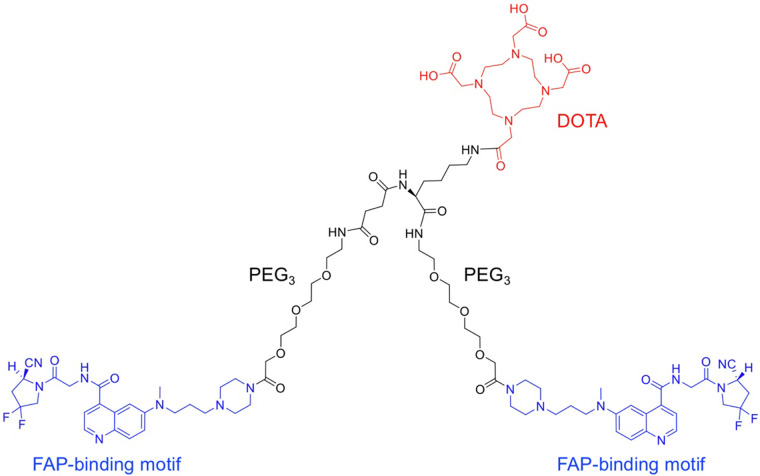
Chemical structure of DOTA-2P(FAPI)_2_.

The dimer of FAPI-46 with 2 PEG_3_ groups and the chelator DOTA were synthesized (Fig. 1; Supplemental Fig. 1). Subsequently, 2 radioligands were prepared as controls and tests: ^68^Ga-FAPI-46, first reported by Loktev et al. (3), and ^68^Ga-DOTA-2P(FAPI)_2_, the dimer of FAPI-46. ^68^Ga-DOTA-2P(FAPI)_2_ and ^68^Ga-FAPI-46 were radiolabeled at an average specific activity of 37 and 16.5 GBq/μmol, respectively, with more than 95% radiochemical purity after purification (Supplemental Figs. 2A and 2B).

The stability of ^68^Ga-DOTA-2P(FAPI)_2_ was evaluated in phosphate-buffered saline and FBS at 1, 2, and 4 h after incubation. High-performance liquid chromatography analysis results showed that ^68^Ga-DOTA-2P(FAPI)_2_ had high stability for up to 4 h, with no significant demetalation observed in either phosphate-buffered saline (92.78%) or FBS (97.80%) (Supplemental Figs. 2C and 2D).

### Cell-Based Experiments

**FIGURE 2.**
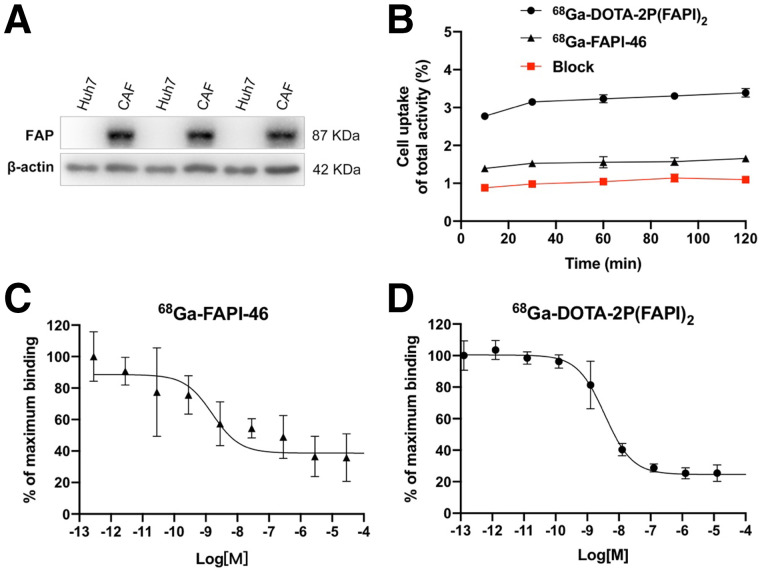
(A) FAP expression in Huh7 cells and CAFs assayed using Western blotting. (B) Cell uptake assay of ^68^Ga-DOTA-2P(FAPI)_2_, ^68^Ga-FAPI-46, and blocking experiment on CAFs (*n* = 3). (C) Inhibition of ^68^Ga-FAPI-46 binding to FAP on CAFs by unlabeled FAPI-46 (2.83 × 10^−4^ to 10^−13^ M; *n* = 3). (D) Inhibition of ^68^Ga-DOTA-2P(FAPI)_2_ binding to FAP on CAFs by unlabeled FAPI-46 (1.27 × 10^−4^ to 10^−13^ M; *n* = 3).

The binding properties of FAPI variants were first verified and evaluated in a FAP-expressing cell line. Western blotting results revealed that FAP was highly expressed in CAFs and negatively expressed in Huh7 cells (Fig. 2A). Since FAP is expressed on the CAF surface, ^68^Ga-DOTA-2P(FAPI)_2_ and ^68^Ga-FAPI-46 could bind to FAP rapidly. Uptake of ^68^Ga-FAPI-46 reached approximately 1.5% after 10 min of incubation and then slightly increased until 120 min. The uptake pattern of ^68^Ga-DOTA-2P(FAPI)_2_ was similar; however, the uptake value was approximately double. Regarding the cell uptake–blocking experiment, the FAPI-46 precursor could significantly block binding between ^68^Ga-DOTA-2P(FAPI)_2_ and FAP (Fig. 2B). The mean ± SD and error of the inhibitory concentrations of 50% were 2.06 ± 1.84 nM and 1.06 nM, respectively, for the monomer and 3.68 ± 1.82 nM and 1.05 nM, respectively, for the dimer (Figs. 2C and 2D). The comparable inhibitory concentrations of 50% for DOTA-2P(FAPI)_2_ and FAPI-46 suggest that the dimerization of the FAPI structure has a minimal effect on the receptor-binding avidity.

### HCC-PDX Establishment and Validation

Two different groups of HCC-PDXs were successfully established, denoted as HCC-PDX-1 and HCC-PDX-2. The 2 groups had different clinical data (patient 1: male, T1aN0M0, poorly differentiated HCC; patient 2: male, T1bN0M0, moderately differentiated HCC), both with high levels of FAP expression (Supplemental Fig. 3). Both groups showed histopathologic characteristics (FAP, Ki-67, and hematoxylin and eosin; Supplemental Fig. 3) consistent with their corresponding primary HCC and were chosen as the experimental models to evaluate the in vivo behavior of ^68^Ga-DOTA-2P(FAPI)_2_.

### Small-Animal PET Studies

**FIGURE 3.**
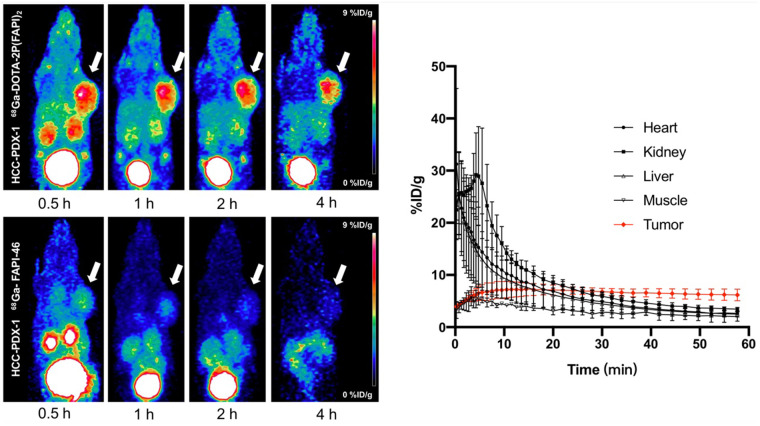
Representative static PET imaging of ^68^Ga-DOTA-2P(FAPI)_2_ (left top) and ^68^Ga-FAPI-46 (left bottom) in HCC-PDX-1, and dynamic time–activity curves of ^68^Ga-DOTA-2P(FAPI)_2_ (right) in heart, kidney, liver, muscle, and tumor tissues.

**FIGURE 4.**
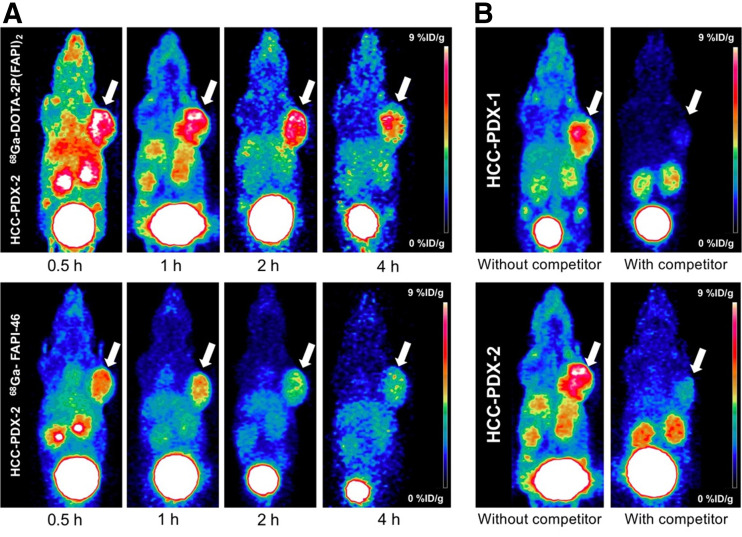
(A) Representative static PET imaging of ^68^Ga-DOTA-2P(FAPI)_2_ and ^68^Ga-FAPI-46 in HCC-PDX-2. (B) Representative static PET imaging of ^68^Ga-DOTA-2P(FAPI)_2_ in HCC-PDX-1 and HCC-PDX-2 with and without simultaneous injection of unlabeled FAPI-46 as competitor 1 h after administration.

In HCC-PDX-1, both radiotracers were absorbed strongly by the tumor at 0.5 h after injection, and the uptake decreased relatively slowly until 4 h (Fig. 3). However, tumor uptake of ^68^Ga-DOTA-2P(FAPI)_2_ was significantly higher than that of ^68^Ga-FAPI-46. The detailed percentage injected dose (%ID)/g in tumor for both tracers from small-animal PET are shown in Supplemental Figure 4A. Other organs demonstrated low nonspecific binding that quickly decreased (Supplemental Figs. 4B and 4E), resulting in a low background signal and favorable tumor-to-background ratios. For a comprehensive investigation of the early pharmacokinetics of ^68^Ga-DOTA-2P(FAPI)_2_, 60-min dynamic PET was performed on HCC-PDX-1. The tumor accumulation of ^68^Ga-DOTA-2P(FAPI)_2_ was rapid, and the time dependency of the FAPI dimer uptake was similar to that of other FAPI tracers. In contrast, the heart, kidney, and liver uptake showed sharp elimination (Fig. 3). Regarding ^68^Ga-DOTA-2P(FAPI)_2_ PET in HCC-PDX-2, tumor accumulation was rapid. Slightly decreased tumor uptake was observed from 30 min to 1 h, and it then remained constant between 1 and 4 h (Fig. 4A; Supplemental Fig. 5), similarly to that in HCC-PDX-1. The 60-min dynamic PET was also performed on HCC-PDX-2 (Supplemental Fig. 6).

Target specificity was evaluated by simultaneous administration of unlabeled FAPI-46 as a competitor with ^68^Ga-DOTA-2P(FAPI)_2_. Tumor uptake 1 h after injection was greatly suppressed by blocking in HCC-PDX-1 and HCC-PDX-2, and radiotracer clearance in most organs was faster than that without blocking (Fig. 4B). The uptake values of the tumor and key organ with or without competitor are presented in Supplemental Figures 4F and 5F.

### Organ Distribution in HCC-PDX-1

**FIGURE 5.**
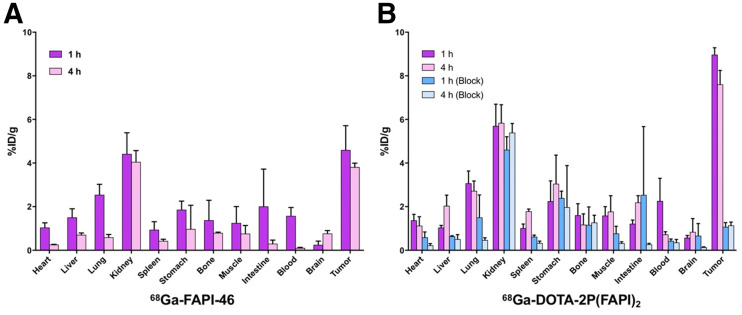
(A) Ex vivo biodistribution of ^68^Ga-FAPI-46 in HCC-PDX-1, 1 and 4 h after injection (*n* = 3/group). (B) Ex vivo biodistribution of ^68^Ga-DOTA-2P(FAPI)_2_ in HCC-PDX-1, 1 and 4 h after injection, with and without coadministration of unlabeled FAPI-46 as blocking agent (*n* = 3/group).

The biodistribution of ^68^Ga-FAPI-46 in HCC-PDX-1 was determined by ex vivo counting in tissues collected 1 and 4 h after injection (Fig. 5A). At 1 h after injection, ^68^Ga-FAPI-46 accumulated mainly in the tumor (4.60 ± 1.12 %ID/g) and kidney (4.42 ± 0.97 %ID/g), and the tumor-to-kidney ratio was 1.05 ± 0.18. Four hours after injection, ^68^Ga-FAPI-46 in the blood, heart, liver, lung, and spleen decreased sharply, whereas tumor uptake was steady (3.81 ± 0.18 %ID/g).

The biodistribution of ^68^Ga-DOTA-2P(FAPI)_2_ was also assessed in the same PDX model by comparison (Fig. 5B). Consistently with the PET findings, ^68^Ga-DOTA-2P(FAPI)_2_ demonstrated higher uptake in the tumor than did ^68^Ga-FAPI-46, 1 h after injection (8.97 ± 0.32 vs. 4.60 ± 1.12 %ID/g, *P* = 0.003) and 4 h after injection (7.61 ± 0.64 vs. 3.81 ± 0.18 %ID/g, *P* = 0.001). The organ uptake of ^68^Ga-DOTA-2P(FAPI)_2_ was slightly greater than that of ^68^Ga-FAPI-46 both 1 and 4 h after injection. As a result, ^68^Ga-DOTA-2P(FAPI)_2_ had a higher tumor-to-kidney ratio than did ^68^Ga-FAPI-46 (1.60 ± 0.26 vs. 1.05 ± 0.18, *P* = 0.039, 1 h after injection), although the difference was not significant 4 h after injection (1.33 ± 0.29 vs. 0.95 ± 0.09, *P* = 0.093).

Regarding the blocking group, a dramatic decrease in radioactivity was detected in most organs (Fig. 5B), and the tumor uptake decreased most significantly (8.97 ± 0.32 vs. 1.07 ± 0.19 %ID/g 1 h after injection, *P* < 0.001, Student *t* test; 7.61 ± 0.64 vs. 1.14 ± 0.15 %ID/g 4 h after injection, *P* = 0.002, Student *t* test).

Additional biodistribution and PET studies were performed to rule out the effect of molar activity on comparative experiments between FAPI dimer and FAPI-46. The amount of precursor administered was 23 μg (25.9 nmol) for FAPI-46 and 50 μg (25.3 nmol) for DOTA-2P(FAPI)_2_, resulting in the same specific activity for ^68^Ga-FAPI-46 and ^68^Ga-FAPI dimer. Under these circumstances, the results from the biodistribution study demonstrated that ^68^Ga-FAPI dimer had higher tumor uptake than did ^68^Ga-FAPI-46 (8.45 ± 2.19 vs. 4.03 ± 0.69 %ID/g; *P* = 0.029, Student *t* test, Supplemental Fig. 7). Similar results were observed from the PET imaging study (Supplemental Fig. 8).

### Adverse Events

All observed vital signs (including blood pressure, heart rate, and body temperature) remained normal during the injection and at the 4-h follow-up. No individuals reported any adverse events.

### Dosimetry Estimate

**FIGURE 6.**
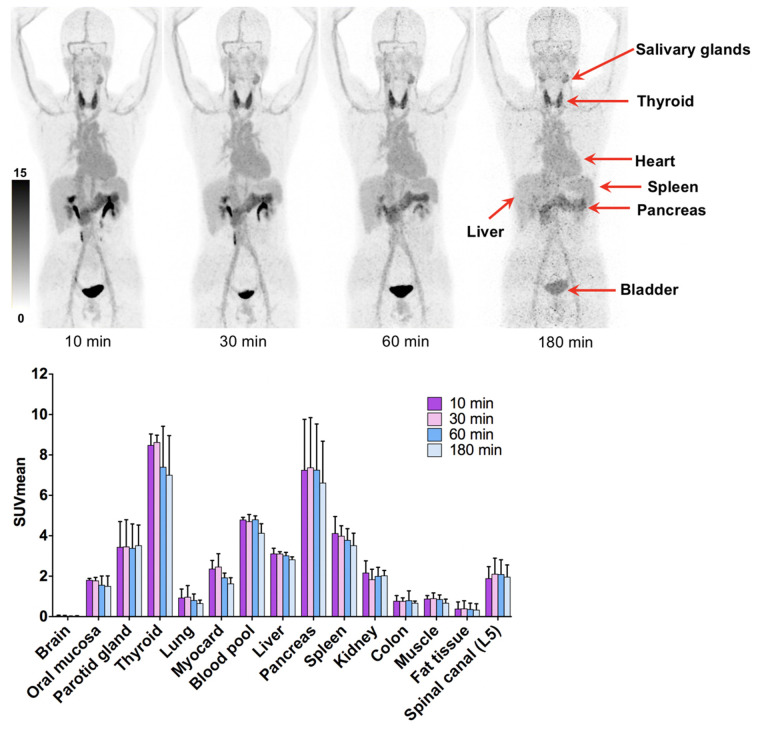
^68^Ga-DOTA-2P(FAPI)_2_, 10, 30, 60, and 180 min after injection, in healthy volunteers (top), and SUV_mean_ of healthy organs at different time points (bottom).

The dosimetry reports and a representative figure for 3 healthy volunteers are shown in Table 1 and Figure 6, respectively. There was no time dependency of tracer uptake, showing that the tracer distribution was not obviously changing after 10 min. The effective dose of ^68^Ga-DOTA-2P(FAPI)_2_ was 1.19E−02 mSv/MBq, calculated using OLINDA. The organ with the highest effective dose was the thyroid (3.11E−03 mSv/MBq), followed by the liver (1.65E−03 mSv/MBq) and lungs (1.36E−03 mSv/MBq). Overall, the effective dose of ^68^Ga-DOTA-2P(FAPI)_2_ was comparable to the effective doses of ^68^Ga-FAPI-02 (1.80E−02 mSv/MBq) and ^68^Ga-FAPI-04 (1.64E−02 mSv/MBq) (4) and higher than the effective dose of ^68^Ga-FAPI-46 (7.80E−03 mSv/MBq) (23).

**TABLE 1.** ^68^Ga-DOTA-2P(FAPI)_2_ Dosimetry Summary of Effective Doses Using OLINDA/EXM, Version 1.1

Target organ	Mean (mSv/MBq)	SD (mSv/MBq)
Adrenal glands	7.98E−05	3.04E−05
Brain	3.16E−05	1.96E−05
Breasts	6.36E−05	1.09E−05
Gallbladder wall	—	—
Lower large intestine wall	9.22E−04	2.33E−04
Small intestine	5.18E−05	1.84E−05
Stomach wall	7.12E−04	3.16E−05
Upper large intestine wall	2.79E−05	1.13E−05
Heart wall	—	—
Kidneys	1.01E−04	4.96E−05
Liver	1.65E−03	4.12E−04
Lungs	1.36E−03	4.59E−04
Muscle	4.19E−05	2.07E−05
Ovaries	5.94E−04	1.06E−04
Pancreas	7.61E−04	8.05E−04
Red marrow	1.12E−03	1.33E−04
Osteogenic cells	6.47E−05	6.87E−06
Skin	1.22E−05	2.32E−06
Spleen	1.58E−04	9.12E−05
Thymus	9.64E−06	4.47E−06
Thyroid	3.11E−03	4.68E−04
Urinary bladder wall	1.04E−03	5.22E−04
Uterus	1.27E−05	6.00E−06
Effective dose equivalent	1.69E−02	1.92E−03
Effective dose	1.19E−02	9.45E−04

### ^68^Ga-DOTA-2P(FAPI)_2_ PET Imaging in Cancer Patients

**FIGURE 7.**
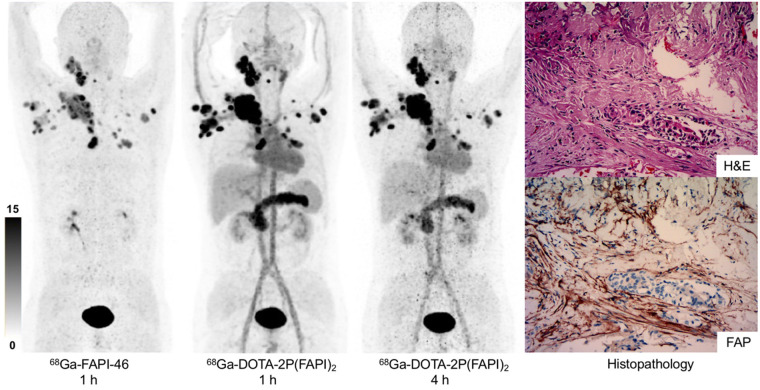
^68^Ga-FAPI-46, 1 h after injection, and ^68^Ga-DOTA-2P(FAPI)_2_, 1 and 4 h after injection, in patient with metastatic thyroid cancer. Hematoxylin and eosin (H&E) staining and FAP immunohistochemistry staining showed high FAP expression in tumor stroma (×100).

^68^Ga-FAPI-46 and ^68^Ga-DOTA-2P(FAPI)_2_ PET/CT scans were performed after 60 min of intravenous administration in 3 patients: one with nasopharyngeal nonkeratinized undifferentiated carcinoma and wild diffuse bone metastases after chemoradiotherapy and immunotherapy; one with papillary thyroid carcinoma and wild diffuse lymph node metastases after total thyroidectomy and multiple cycles of radioiodine treatment; and one with HCC, who was treatment-naïve. Representative PET images of these 3 patients after administration of ^68^Ga-FAPI-46 and ^68^Ga-DOTA-2P(FAPI)_2_ are shown in Figure 7 and Supplemental Figures 9 and 10. In the patient with metastatic thyroid cancer, ^68^Ga-DOTA-2P(FAPI)_2_ accumulated mainly in the tumor, pancreas, submandibular glands, and blood pool. Interestingly, the activity of FAPI dimer in the blood pool remained at a high level (SUV_max_, 8.3) 4 h after injection. All tumor lesions were clearly visible because of the favorable tumor-to-background ratios. In the lesion-to-lesion comparison, the dimer uptake in 21 lesions (from 3 patients) was higher than monomer uptake (SUV_max_ 1 h after injection, 8.1–39.0 vs. 1.7–24.0, respectively; *P* < 0.001 by Wilcoxon matched-pairs signed-rank test; mean SUV_max_, 15.3 vs. 23.9; Supplemental Table 1). In addition, the ^68^Ga-2P(FAPI)_2_ uptake in tumors was slightly decreased from 1 to 4 h (SUV_max_ 1 h after injection, 8.1–39.0; SUV_max_ 4 h after injection, 6.6–35.0).

## DISCUSSION

With a burst of preclinical and clinical research on quinoline-based FAPI variants, 2 main hurdles remain: improving the tumor retention time and finding the appropriate preclinical models. Since FAP is overexpressed mainly in CAFs and not in tumor cells, the tumor cell-line transfected with human or murine FAP could not reflect the tumor microenvironment (3*,*^7^). In contrast, PDXs can reliably reproduce a patient’s parental tumor for histopathology and genetics (15). Therefore, PDXs are suitable models for studying tumor biology, including the microenvironment and patient sensitivity to target agents. Despite an increasing number of case studies (6–9) and 2 clinical trials with a small patient population (13*,*^24^) for FAP-based PTRT, basic research on this topic is rare (10). In the present study, PDXs derived from HCC could maintain the principal histopathologic characterization of the human tumor, confirming the robustness of this model for testing the properties of the new FAPI variant.

With FAPI being a pan-cancer target, labeling of FAPI monomers with different imaging isotopes has shown impressive results in several tumor diagnoses (3–5*,*^25^); however, the pharmacokinetics with fast clearance from blood and short retention in tumors are problematic for PTRT application. Thus, structural modification of FAPI for optimizing tumor uptake and tumor retention time for PTRT is another key research direction.

On the basis of the polyvalent effect, multimeric peptides can help improve tumor-targeting efficacy and generate higher-quality in vivo imaging. This strategy has been widely used in the development of multimeric Arg-Gly-Asp (RGD) peptides (16*,*^17^). Indeed, given that the distance between 2 FAPI motifs in DOTA-2P(FAPI)_2_ may not be long enough, it is unlikely that they would bind to 2 adjacent FAP sites simultaneously. However, the binding of one FAPI motif to FAP will significantly increase the local concentration of a second FAP motif in the vicinity of FAP sites. The locally enhanced FAPI concentration may explain the increased tumor uptake of radiolabeled FAPI dimers compared with their monomeric analogs. Similar findings were observed in the studies of radiolabeled RGD dimers (26). Nonetheless, although tetrameric and octameric peptides possess higher receptor-binding affinity and higher tumor uptake than their dimeric and monomeric counterparts, they also have substantially higher background activity, especially in the kidney (27). Therefore, dimeric peptides seem to be an optimal choice because of their increased tumor uptake and favorable pharmacokinetics (27). PEGylation is another widely used strategy to improve the in vivo pharmacokinetics of radiotracers. According to previous reports of studies in which PEGylated RGD peptides were labeled with different isotopes, PEGylation improved the labeling yield and in vivo pharmacokinetics (16*,*^18^). However, PEGylation also induces hydrophilicity and increases kidney uptake, partially explaining the high initial kidney uptake compared with other FAPI derivatives. In this study, we designed and synthesized a novel FAPI dimer with 2 mini-PEG spacers between the FAPI motifs in homodimeric peptides. The in vitro binding assays demonstrated that DOTA-2P(FAPI)_2_ had specific and high binding affinity to FAP expressed on CAFs, revealing that the polyvalent strategy did not compromise its FAP-binding affinity.

After radiolabeling with ^68^Ga, the FAPI dimers exhibited improved in vivo pharmacokinetics and enhanced tumor uptake compared with the FAPI monomer. Dynamic PET scans in the 2 HCC-PDX groups showed prominent tumor uptake and predominant organ clearance. After the tracers were applied to static PET scans, ^68^Ga-DOTA-2P(FAPI)_2_ demonstrated higher tumor uptake than ^68^Ga-FAPI-46 at all time points examined in both PDXs. In the small-animal PET imaging study, higher initial (30 min after injection) kidney and liver uptake was observed for ^68^Ga-DOTA-2P(FAPI)_2_ than for FAPI-monomers, including ^68^Ga-FAPI-04, ^68^Ga-FAPI-46, and ^18^F-FGlc-FAPI (14). However, the kidney and liver uptake was quickly eliminated at 60 min after injection in PET imaging and the biodistribution study. The high initial kidney uptake and rapid renal clearance may be attributed to the insertion of 2 PEG groups, which improved the hydrophilic properties (16*,*^18^). Nevertheless, the main organ uptake should be carefully estimated for safety-dose limitations when FAPI dimer is labeled with ^177^Lu for targeted radionuclide therapy. The FAP specificity of ^68^Ga-DOTA-2P(FAPI)_2_ was strongly confirmed by effective uptake inhibition in the presence of unlabeled FAPI-46 in cell-uptake, PET, and biodistribution experiments.

It was reported that the FAP-blocking dose (cold mass of FAPI) in one mouse was 30 nmol (7*,*^11^). Since the specific activity of FAPI dimer and FAPI-46 was 37 GBq/μmol and 16.5 GBq/μmol, respectively, in this study, a dose of 7.4 MBq of ^68^Ga-FAPI-46 (0.45 nmol, hot and cold mass) or ^68^Ga-FAPI dimer (0.2 nmol, hot and cold mass) per mouse for PET imaging may have minimal impact on tumor uptake. Therefore, there is no effect of the different injected cold mass of the radiotracers that might have caused the significant differences in tumor uptake. Moreover, additional PET imaging and biodistribution experiments have been performed to rule out the effect of molar activity on all comparative experiments of dimeric and monomeric inhibitors.

The encouraging results of the in vitro and mouse studies led to the clinical translation of FAPI dimer into human subjects. The radiation dose deposition of ^68^Ga-DOTA-2P(FAPI)_2_ in healthy organs was estimated using the PET data of 3 healthy volunteers at 4 time points. The average effective whole-body dose was 1.19E−02 mSv/MBq. This estimate is comparable to the previously reported effective doses of ^68^Ga-FAPI-02 and ^68^Ga-FAPI-04 (1.80E−02 and 1.64E−02 mSv/MBq, respectively), and higher than that of ^68^Ga-FAPI-46 (7.80E−03 mSv/MBq) (4*,*^23^).

Regarding clinical diagnosis, ^68^Ga-DOTA-2P(FAPI)_2_ PET/CT imaging in the patients showed a rapid and stable accumulation of the dimer in tumorous lesions, consistent with the results of animal experiments. Tumor uptake in most lesions was significantly higher with ^68^Ga-DOTA-2P(FAPI)_2_ than with ^68^Ga-FAPI-46, leading to visualization of primary lesions and metastases more clearly. Interestingly, retention of the tracer in the patient blood pool remained high 4 h after injection, in contrast to mouse findings. The prolonged retention in the blood pool may make DOTA-2P(FAPI)_2_ an attractive tracer for PTRT applications. Although the PET/CT results were encouraging in patients, high physiologic uptake in the thyroid and pancreas should be noted. Nevertheless, DOTA-2P(FAPI)_2_ labeling with ^68^Ga demonstrated favorable data in cells, mice, and patients. For future development, especially for antitumor therapeutic applications, labeling of the ligand with therapeutic radionuclides such as ^177^Lu and ^90^Y should be considered to compare the FAPI dimer with the monomer.

## CONCLUSION

^68^Ga-DOTA-2P(FAPI)_2_ provides better tumor uptake and longer tumor retention than does ^68^Ga-FAPI-46 and could be a promising tracer for both diagnostic imaging and targeted radionuclide therapy in malignant tumors with positive FAP expression. Further work to optimize the pharmacokinetics of DOTA-2P(FAPI)_2_ and evaluate its antitumor efficacy after labeling with therapeutic isotopes should be envisaged.

## DISCLOSURE

This work was funded by the National Natural Science Foundation of China (grants 82071961, 81901805, and 81772893) and by the key medical and health projects in Xiamen (grant 3502Z20191104). No other potential conflict of interest relevant to this article was reported.

## Notes

KEY POINTS **QUESTION:** How can tumor uptake and retention of the FAP-targeting molecular agents be optimized? **PERTINENT FINDINGS:** In a pilot clinical cancer imaging study, ^68^Ga-DOTA-2P(FAPI)_2_ was synthesized as a FAPI dimer and tested for its pharmacokinetic properties. Its tumor uptake was higher than that of monomeric FAPIs in vitro, in vivo, and in cancer patients. **IMPLICATIONS FOR PATIENT CARE:** Because ^68^Ga-DOTA-2P(FAPI)_2_ shows improved tumor uptake and retention properties, it could be a promising candidate tracer for both diagnostic imaging and targeted therapy of malignant tumors with positive FAP expression.
